# Stereology in Grading and Prognosis of Canine Cutaneous Mast Cell Tumors

**DOI:** 10.1177/0300985820985138

**Published:** 2021-02-12

**Authors:** Mafalda Casanova, Sandra Branco, Inês Berenguer Veiga, André Barros, Pedro Faísca

**Affiliations:** 1 70904 Instituto Gulbenkian de Ciência, Oeiras, Portugal; 2 70989 Universidade de Évora, Évora, Portugal; 3 54179 University of Bern, Bern, Switzerland; 4 FMV-ULHT, Lisbon, Portugal; 5 DNATech, Lisbon, Portugal

**Keywords:** dogs, mast cell tumor, mean nuclear volume, skin, stereology, histological grading, prognosis, surgical pathology

## Abstract

Canine cutaneous mast cell tumors (ccMCTs) are currently graded according to Patnaik and Kiupel grading schemes. The qualitative and semiquantitative parameters applied in these schemes may lead to inter- and intraobserver variability. This study investigates the prognostic value of volume-weighted mean nuclear volume ($\bar{v_{v}}$), a stereological estimation that provides information about nuclear size and its variability. $\bar{v_{v}}$ of 55 ccMCTs was estimated using the “point-sampled intercept” method and compared with histological grade and clinical outcome. The clinical history of dogs treated with surgical excision alone was available for 30 ccMCTs. Statistical differences in $\bar{v_{v}}$ were found between grade II ($\bar{x}\;$= 115 ± 29 µm^3^) and grade III ccMCTs ($\bar{x\;}$= 197 ± 63 µm^3^), as well as between low-grade ($\bar{x\;}$= 113 ± 28 µm^3^) and high-grade ccMCTs ($\bar{x}\;$= 184 ± 63 µm^3^). An optimal cutoff value of $\bar{v_{v}}$ ≥ 150 µm^3^ and $\bar{v_{v}}$ ≥ 140 µm^3^ was determined for grade III and high-grade ccMCTs, respectively. In terms of prognosis, $\bar{v_{v}}\;$ was not able to predict the clinical outcome in 42% of the cases; however, cases with $\bar{v_{v}}\;$<125 µm^3^ had a favorable outcome. These results indicate that, despite having limited prognostic value when used as a solitary parameter, $\bar{v_{v}}$ is highly reproducible and is associated with histological grade as well as with benign behavior.

Canine cutaneous mast cell tumors (ccMCTs) are among the most frequently diagnosed skin tumors in dogs.^24,41,43^ These tumors display a variable and unpredictable biological behavior, ranging from benign behavior to potentially fatal metastatic tumors.^13,27^ Tumor grade is widely used for prognostication;^4,13,25^ however, no single parameter can accurately predict the biological behavior of ccMCTs. Several additional prognostic factors have been studied to improve ccMCT prognostication, including cytological grade,^6,29^ expression pattern of KIT protein,^14^ detection of c-KIT mutations,^40,42^ proliferation markers,^41,43^ and margin evaluation.^29,30^

Most histological grading schemes rely on the subjective evaluation of morphologic and cytological parameters, which are prone to personal bias and often lead to intra- and interobserver variability.^1,28^ In the case of ccMCTs, the most frequently used grading systems are those of Patnaik and Kiupel. The Patnaik system grades ccMCTs as grade I (G1), II (G2), and III (G3) according to the degree of mast cell differentiation, morphology, cellularity, extent of tissue involvement, stromal reaction, and mitotic count.^25^ One of the problems with this grading system is the pronounced interobserver variability, particularly among the grading of G1 and G2 ccMCTs.^13,22,23,27,38,40^ To overcome the subjectivity associated with Patnaik grading, a 2-tier grading scheme was developed by Kiupel et al.^13^ The latter grades ccMCTs as low-grade (LG) or high-grade (HG) based on semiquantitative parameters, such as number of mitotic figures, multinucleated nuclei and bizarre nuclei per 10 hpf, and the presence of karyomegaly.^13^ Several studies have shown the use of Kiupel grading not only significantly decreases interobserver variation but also has a superior prognostic value.^13,27,40,41^

This study investigated the advantage of quantifying nuclear size and its variability to overcome the subjectivity associated with ccMCT histological grading. Previous measures of nuclear size have been studied in ccMCT cytological and histological samples.^18,38,39^ These studies found associations between either ccMCT nuclear area or perimeter and Patnaik grade. Interestingly, statistical differences were found between G1 and G3 ccMCTs, as well as between G2 and G3, but not between G1 and G2. Two-dimensional morphometrical estimates have the disadvantage that the position and orientation of a section plane across a 3-dimensional object influence the size, shape, and frequency of the 2-dimensional profile.^5,19^ Consequently, although there is a correlation between nuclear area or perimeter and the real nuclear size, the true relation between these parameters is uncertain, no matter how elegant the statistical model^33^ (Fig. 1). Design-based stereological methods, on the other hand, are based on statistical sampling and geometrical principles to recover 3-dimensional information from 2-dimensional sections. These methods eliminate assumptions about the object’s shape and orientation, thus allowing for precise and reproducible measures of numerous parameters, including nuclear size.^5,19,28^ Stereological measures of nuclear size are best performed with the “point-sampled intercept” (PSI) method, which provides a measure of volume-weighted mean nuclear volume ($\bar{v_{v}})$.^11^ $\;\;\bar{v_{v}}\;$ is volume-weighted, meaning that nuclei are sampled in proportion to their volume, thus larger nuclei have greater probability of being sampled. This measure is therefore a reflection of nuclear size and pleomorphism, increasing not only as nuclei enlarge but also with nuclear size variability.^9,33^ The PSI method has been used not only in malignancy grading of solid tumors,^12,15–17,21,28,34,35,37^ but also in mean particle volume estimation of normal histological structures, such as pancreatic islets and alveoli.^2,3,32^ This study estimates $\bar{v_{v}}$ in ccMCTs and compares it with histological grade and clinical outcome.

**Figure 1.**
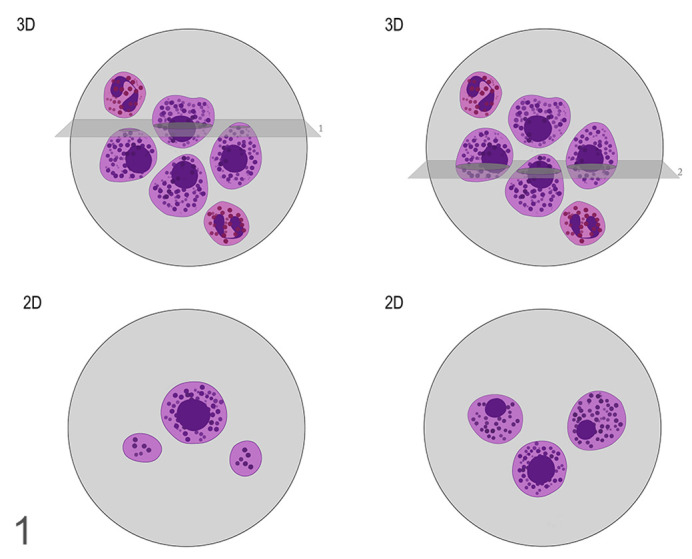
Two-dimensional representations of sections across 3-dimensional mast cells. The orientation of the slice influences not only the frequency with which nuclei are sampled, but also their dimension. Although there is a relation between the nuclear profile area and its true size, this relation is uncertain due to the loss of 3-dimensional information. The right and left panels represent 2 different sections through that same cell population. The top panels are 3-dimensional representations of the cells showing the location of slices. The lower panels are the appearances of the resulting 2-dimensional sections.

## Materials and Methods

### Case Selection and Histopathology

Fifty-five paraffin-embedded ccMCTs diagnosed in 2017 were selected for this study from the archives of DNATech, Lisbon, Portugal. These cases were selected if the diagnosis had been made at least 1 year before the start of the study (May 2019). Three-micrometer sections were routinely processed and stained with hematoxylin and eosin for tumor grading and stereological estimates. Each tumor was blindly graded according to the Patnaik and Kiupel grading schemes by 3 experienced pathologists (IBV—ECVP board certified, and SB and PF—professors of veterinary pathology with more than 20 years experience). The final diagnosis was established by the consensus of at least 2 observers. Histological grade was assigned according to the criteria described in the original papers.^14,25^

### Outcomes

Follow-up of dogs treated with surgical excision alone was available for 30 cases. The veterinary clinicians provided clinical data regarding existence of postsurgical local recurrence, metastasis, and/or mast cell tumor-related death (including euthanasia).

The minimum follow-up period was 1 year. Dogs with postsurgical resolution of disease were given an outcome value of 0 (OC0), whereas outcome value of 1 (OC1) included cases that died or were euthanized as a result of local recurrence or development of nodal or visceral metastasis. The lateral and deep surgical margins (cm) were also evaluated at the time of submission and compared between groups.

### Stereology

$\bar{v_{v}}$ was estimated in one section per tumor, produced by sectioning the tumor perpendicularly to the epidermis.^10,36^ Measurements were made on the same slide previously used for diagnostic evaluation and tumor grading^9^.

Whole-slide images were obtained (NanoZoomer-SQ Digital slide scanner, Hamamatsu Photonics) with 40× resolution and 800× magnification. Measurements were performed on newCAST stereological software (Visiopharm). In each slide, the region of interest (ROI), that is, the total area of the tumor, was delineated manually at low magnification. Within each ROI, fields of view (1000×, *A* = 10300.84 µm^2^) were automatically selected in a systematic random fashion by the software. According to Gundersen and Jensen,^11^ approximately 75 nuclei per tumor are needed to accurately estimate $\;\bar{v_{v}}$, ranging from 50 to 100 nuclei. The number of fields of view required was influenced by the tumor’s cellularity. Fields of poor focus with indistinguishable nuclear borders were excluded from measurement.

**Figure 2.**
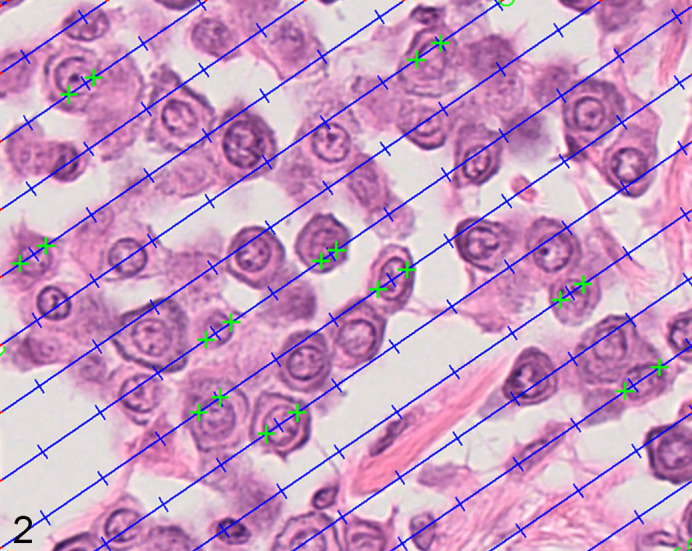
Estimation of mean nuclear volume using the point-sampled intercept method. Fields of view from a mast cell tumor are automatically generated with a constant step size. The nuclear profiles are sampled with points (blue hash marks) and the associated test-lines create linear intercepts across these profiles. The length of the intercepts is traced by marking the nuclear borders in the direction of the line (green hash marks). The goal is to measure ∼75 intercepts per tumor.

$\bar{v_{v}}$ of ccMCTs was estimated according to the PSI method, in which the nuclei are sampled with probes of test-lines and associated points, superimposed onto the fields of view.^11^ Every time a nucleus is hit by a point (+), the associated test-line creates an intercept across the nuclear profile, whose length ($l_{0}^{3}$) is measured by marking the nuclear borders (see Fig. 2). These lines were randomly rotated between fields, allowing randomizing of the nuclei and orientation in which these were measured.^11^ In total, 4 measurements were performed per tumor (MC—3 measurements, PF—1 measurement).

$\bar{v_{v}}\;$ was given by

$$\bar{v_{v}}\;=\;\frac{\pi }{3n}\overset{n}{\underset{i=1}{\sum }}l_{0,i}^{3}$$

In which $l_{0}^{3}$ is the cubed intercept length (µm^3^) and *n* is the number of intercepts measured.

### Statistical Analysis

Statistical analysis was performed using R software with the DescTools (version 0.99.37),^31^ irr (version 0.81.4),^8^ ggplot2 (version 3.2.1),^44^ and pROC packages (version 1.14.0).^26^

For quantitative variables, such as $\bar{v_{v}}$ measurements, before any hypothesis testing, normality was assessed with Shapiro-Wilk test and homoscedasticity with Levene test; following the obtained results, a parametric or nonparametric approach was selected. For qualitative variables, χ^2^ test or Fisher exact test were used, depending on the number of cases for each category.

Regarding Patnaik and Kiupel grading, the agreement among pathologists was evaluated with Fleiss’ *κ* statistics.^20^

Considering the paired measurements (different measures from the same sample), the differences between $\;\bar{v_{v}}$ measurements (including the average) and the concordance with average were assessed with nonparametric Friedman’s test and the Lin’s concordance correlation coefficient, respectively. To evaluate differences of $\bar{v_{v}}$ between Patnaik and Kiupel grades, a parametric Welch 2-sample *t*-test was used. The ability of $\bar{v_{v}}$ to discriminate histological grades was assessed using receiver operating characteristics (ROC) curve. The association between tumor grade and mortality was investigated by means of Kaplan-Meier curves.

For the variable Outcome, a subset of 30 cases for which we had information on the variables age, sex, breed, and surgical margins was used. To assess if there were any differences between outcome on those variables, as well as differences of $\bar{v_{v}}$ between outcomes, a nonparametric Wilcoxon’s sum-rank test was used.

To determine if the $\bar{v_{v}}$ measurements could be used to efficiently discriminate between outcomes, a 2-step analysis was performed: Using an ROC curve, the ability of $\bar{v_{v}}$ to correctly identify the outcome was assessed. Next, considering the impact that confounding variables can influence the measurements, a multivariate logistic regression was performed using age and sex in addition to $\bar{v_{v}}$. To assess the quality of the overall capability of regressions to explain our data, we used McFadden’s pseudo-*R* ^2^. Additionally, using the cutoff calculated in the ROC curve analysis to stratify the population, survival analysis was performed by means of Kaplan-Meier curves, and the log rank test for comparison of the survival among groups. For all analyses, a value of *P* < .05 was considered as statistically significant.

## Results

**Figures 3–4.**
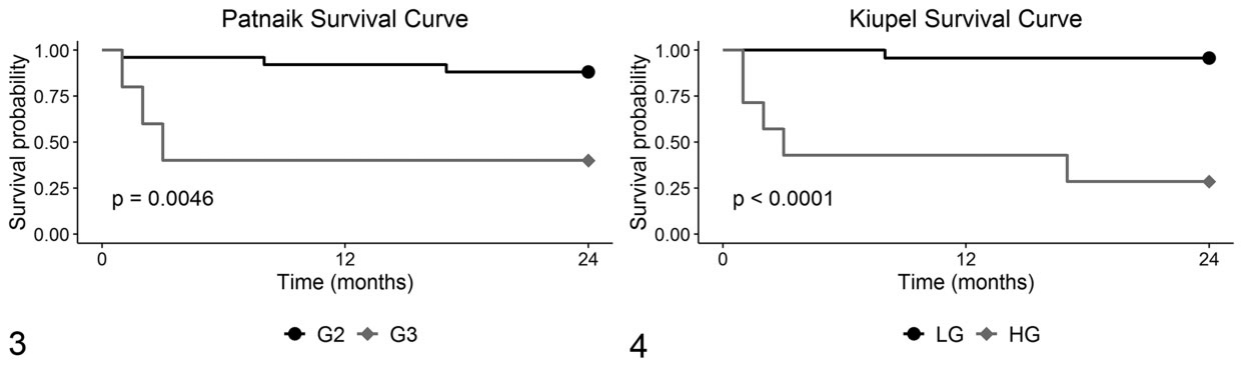
Survival curves for mortality in 30 canine mast cell tumors, graded according to the Patnaik grading system (Fig. 3) and the Kiupel system (Fig. 4). Abbreviations: G2, Patnaik grade 2; G3, Patnaik grade 3; LG, Kiupel low-grade; HG, Kiupel high-grade.

Fifty-five ccMCTs were graded by consensus among 3 pathologists based on Patnaik and Kiupel grading systems. Based on the Patnaik grading system, 39 (71%) ccMCTs were G2 and 16 (29%) as G3. No tumors were diagnosed by consensus as G1, even though 11 tumors were diagnosed as G1 by 1 out of 3 pathologists. The agreement among pathologists was fair in Patnaik grading (*κ* = 0.32), with 44% concordance in the assignment of G2 and 56% concordance in the assignment of G3 (Table 1). G3 ccMCTs were associated with increased mortality and shorter survival time (Fig. 3). Based on the Kiupel grading system, 35 (64%) ccMCTs were diagnosed as LG and 20 (36%) as HG. There was moderate agreement in Kiupel grading (*κ* = 0.46), with 66% concordance in the assignment of LG and 55% concordance in the assignment of HG ccMCTs (Table 1). Overall, LG were graded as G2 and HG were graded as G3, except for 4 HG graded as G2. HG ccMCTs were associated with increased mortality and shorter survival time (Fig. 4).

**Table 1.** Grade Assignment for 55 Canine Cutaneous Mast Cell Tumors by 3 Pathologists^a^. The Data Show the Number and Percentage of Cases.

Patnaik	*n*	Agreement	Disagreement
Grade 2	39	17 (44%)	22 (56%)
Grade 3	16	9 (56%)	7 (44%)
All grades	55	26 (47%)	29 (53%)
Kiupel	*n*	Agreement	Disagreement
Low-grade	35	23 (66%)	12 (34%)
High-grade	20	11 (55%)	9 (45%)
All grades	55	34 (62%)	21 (38%)

^a^ The data show the number and percentage of cases.

**Figures 5–6.**
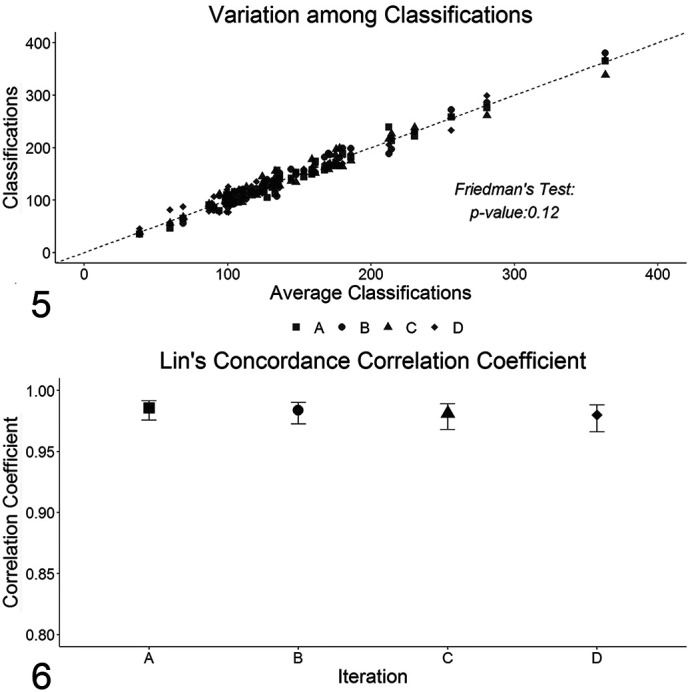
**Figure 5**. Comparison between the values of each individual measurement (*y*-axis) and the average of the 4 measurements (*x*-axis). Each different shape is one measurement. **Figure 6**. Lin’s concordance correlation coefficient between measurements.

Each tumor was measured by 2 observers, totaling 4 measurements per tumor, and each measurement took approximately 10 minutes. To ease the following analysis, the average of those 4 measurements was calculated and compared with the obtained data. There were no statistical differences among measurements including the average (see Fig. 5) with this yielding concordance coefficients very close to 1 for the 4 original measurements (Fig. 6). Therefore, the average of the measurements was used as a proxy of the $\bar{v_{v}}$ values.

**Figures 7–8.**
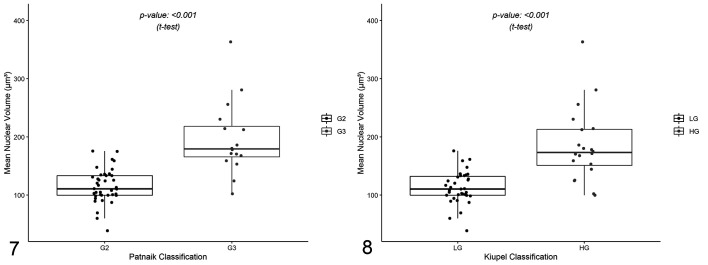
Comparison of the mean nuclear volume values among Patnaik and Kiupel histological grades from 55 cases of canine mast cell tumors. The boxes show first quartile, second quartile (median), and third quartile. The whiskers represent the range (minimum and maximum) values. Mean nuclear volumes are significantly different between grade 2 and grade 3 (Fig. 7), and between low-grade and high-grade (Fig. 8) mast cell tumors (Welch 2-sample *t* test).

**Figures 9–10.**
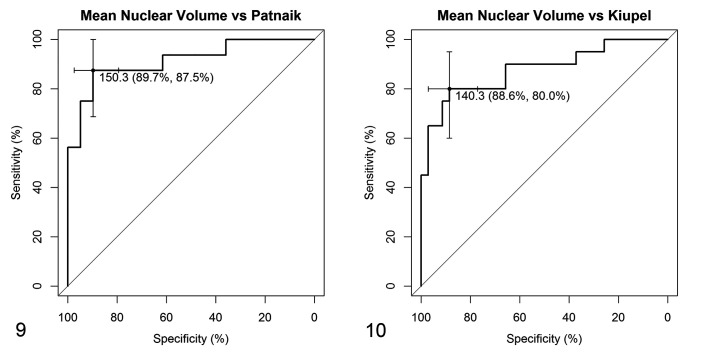
Receiver operating characteristics curves for mean nuclear volume values according to Patnaik (Fig. 9) and Kiupel (Fig. 10) grading systems from 55 cases. For the Patnaik system (Fig. 9), a mean nuclear value ≥150.3 µm^3^ identifies a grade 3 mast cell tumor with 89.7% specificity and 87.5% sensitivity. For the Kiupel system (Fig.10), a mean nuclear value ≥140.3 µm^3^ identifies a high-grade mast cell tumor with 88.6% specificity and 80.0% sensitivity. Abbreviations: G2, Patnaik grade 2; G3, Patnaik grade 3; LG, Kiupel low-grade; HG, Kiupel high-grade.

$\bar{v_{v}}$ was estimated in a total of 55 ccMCTs and ranged from 38.6 to 363.3 µm^3^. $\bar{v_{v}}$ increased significantly with histological grade and statistical differences were found between G2 and G3 ccMCTs (*P* < .001; Fig. 7, Table 2), as well as between LG and HG ccMCTs (*P* < .001; Fig. 8, Table 2). The discriminative power of $\bar{v_{v}}$ between grades was fairly accurate in Patnaik grading (AUC = 91.4%; 95% confidence interval [CI] 82.1% to 100%), while it was less precise in Kiupel grading (AUC = 87.7%; 95% CI 77.3% to 98.1%). In Patnaik grading, a $\bar{v_{v}}$ greater than or equal to 150.3 µm^3^ classified ccMCT as G3 with 89.7% specificity and 87.5% sensitivity (Fig. 9). In Kiupel grading, a $\bar{v_{v}}$ greater than or equal to 140.3 µm^3^ classified a ccMCT as HG with 88.6% specificity and 80.0% sensitivity (Fig. 10).

**Table 2.** Mean Nuclear Volumes (µm^3^) Among Histological Grades of 55 Cutaneous Mast Cell Tumors.

Grade	*n*	Mean	SD	Range	*P* Value
Grade 2	39	115.1	28.8	38.6–175.7	.001
Grade 3	16	196.8	65.5	102.1–363.3	
Low-grade	35	112.7	27.9	38.6–175.7	
High-grade	20	184.7	63.0	99.5–363.1	.001

To access the differences in $\bar{v_{v}}$ between clinical outcomes, 30 cases were selected from our dataset based on the condition that (1) these dogs were treated with surgery alone and (2) follow-up data as well as age, sex, breed, and surgical margins were available. Of these, 17 were females (57%) and 13 were males (43%), including 8 mixed-breed dogs, 6 Labrador Retrievers, 3 Golden Retrievers, 3 French Bulldogs, 2 Boxers, 2 Beagles, 1 Pug, 1 Weimaraner, 1 Basset Hound, 1 Yorkshire Terrier, 1 Bouvier Bernois, and 1 English Bulldog. The mean age at surgical excision was 8 ± 2.9 years, and the follow-up period ranged from 12 to 27 months. During this period, 5 dogs developed an additional ccMCT at the original tumor location and 3 dogs developed ccMCTs at different locations (considered de novo). The OC0 group included 24 dogs that were alive at the end of this study. Of these, 23 had no signs of local or distant recurrence, and one had local recurrence. The OC1 group included 6 dogs that died due to ccMCT-related disease, 5 of which were euthanized. These dogs included 1 case of local recurrence, 1 case of lymph node metastasis, 1 case of distant visceral metastasis, and 3 cases of both local recurrence and distant metastasis. When cytology and histology were not performed, the occurrence of presumptive local recurrence and distant metastasis was based on regrowth of a mass or visceral sonographic alterations.

There were no differences between OC0 and OC1 $\bar{v_{v}}$ according to age, sex, and surgical margins (*P* > .05. Supplemental Table S1). Breed could not be statistically analyzed because there were too many categories.

**Figure 11.**
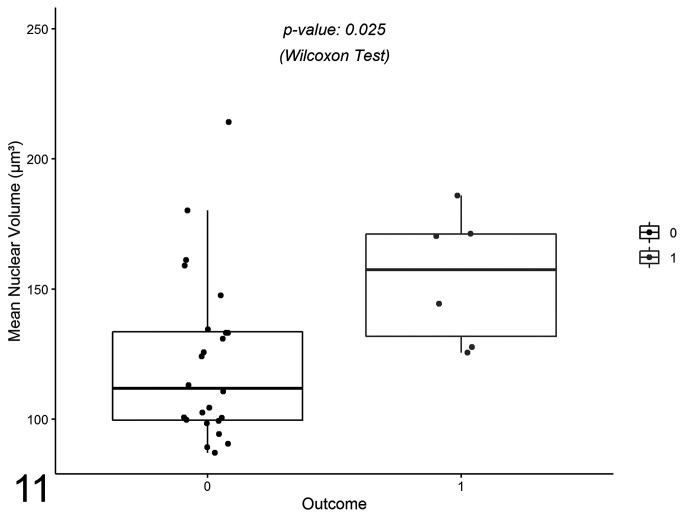
Comparison of the mean nuclear volume values among clinical outcomes from 30 cases of canine mast cell tumor. The boxes show the first quartile, second quartile (median), and third quartile. The whiskers represent the range (minimum and maximum) values. There is a significant difference between outcome OC0 (alive) and OC1 (died) (Wilcoxon’s sum-rank test).

The 24 OC0 cases included 22 G2/LG ccMCTs and 2 G3/HG ccMCTs. The 6 OC1 cases included 1 LG and 5 HG, or 3 G2 and 3 G3. $\bar{v_{v}}$ ranged from 87.1 to 214.2 µm^3^, and was statistically different between OC0 and OC1 (*P* = .025; Fig. 11, Table 3). Although $\bar{v_{v}}$ values overlapped between groups, the 2 cases with highest $\bar{v_{v}}$ of OC0 coincided with the ones graded as G3/HG. Similarly, cases with the lowest $\bar{v_{v}}$ in OC1 group were graded as G2, in spite of a poor outcome (Supplemental Table S1).

**Table 3.** Mean Nuclear Volumes (µm^3^) Among Clinical Outcomes of 30 ccMCTs.

Outcome	*n*	Q1	Median	Q3	Range	*P* Value
OC0	24	99.5	111.8	133.5	87.1–214.1	
OC1	6	131.8	157.4	171.1	125.5–186.0	.025

**Figure 12.**
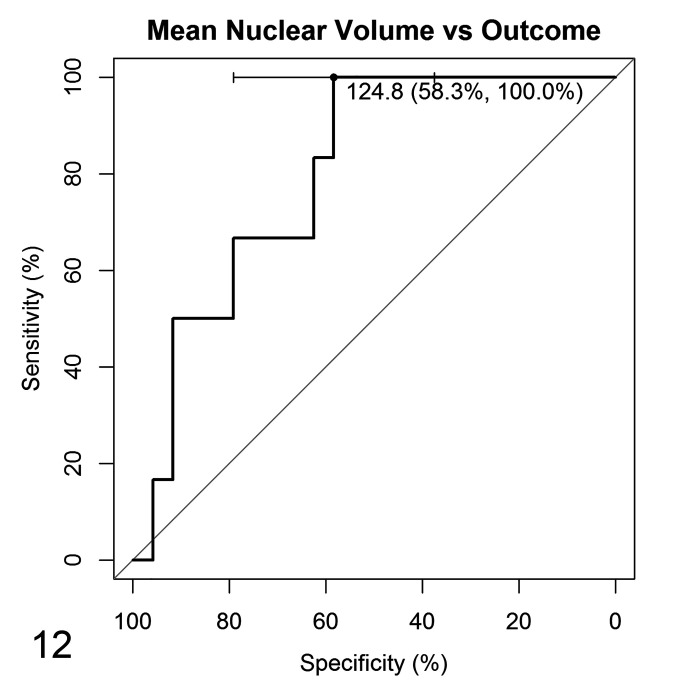
Receiver operating characteristics curve for mean nuclear volume values according to clinical outcome from 30 cases. A mean nuclear value ≥124.8 µm^3^ is associated OC1 (died) with 58.3% specificity and 100% sensitivity.

The potential of $\bar{v_{v}}$ to differentiate the clinical outcomes of these tumors was moderate (AUC = 79.9%; 95% CI 62.5% to 97.2%). A $\bar{v_{v}}$ greater than or equal to 124.8 µm^3^ identified a ccMCT with poor prognosis (OC1 cases) with 100% sensitivity but only 58.3% specificity (Fig. 12).

**Figure 13.**
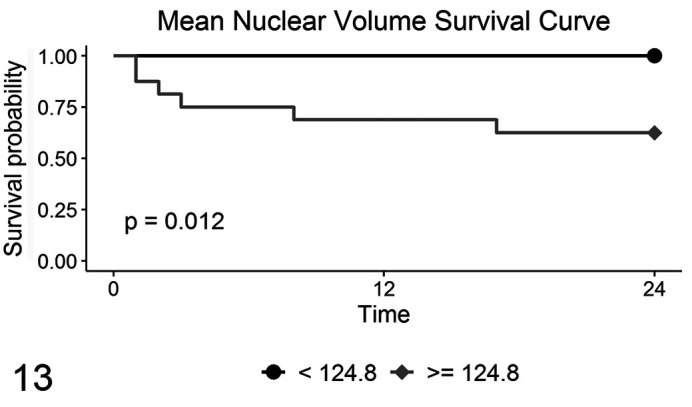
Survival curves for mortality due to mast cell tumors, according to mean nuclear volume values from 30 cases.

By using the cutoff value of 124.8 µm^3^ to separate cases and assessing their mortality, statistically significant differences were observed between cases, with 100% of the animals with $\bar{v_{v}}\;$ below 124.8 µm^3^ alive at the end of the study (Fig. 13). Even though $\bar{v_{v}}$ showed potential to identify benign behavior, the multivariate logistic regression analysis determined that, when age, sex, and margins are considered, $\bar{v_{v}}$ did not show significant capability to predict the outcome, despite the model having McFadden’s pseudo-*R* ^2^ of 0.39.

## Discussion

Our results identified significant variability among pathologists in ccMCT grading. As expected, there was greater consistency in Kiupel grading,^13,27,40,41^ and greater inconsistency in Patnaik grading particularly in the assignment of G2. These results are in accordance with previous studies; however, the concordance between pathologists was lower in this study.^13,27,40^ Although G1 was assigned to 11 tumors by 1 of 3 pathologists, no ccMCTs were graded G1 by consensus in this study. However, G1 ccMCTs are composed of monomorphic, well-differentiated mast cells, hence these tumors would, in theory, have lower $\bar{v_{v}}$ values than G2 ccMCTs. Previous morphometric studies did not find significant differences between G1 and G2 ccMCTs nuclear perimeter and nuclear area.^18,38,39^ Regardless, G1 ccMCTs should be included in future larger studies of $\bar{v_{v}}$.

The PSI method allowed the measurement of $\bar{v_{v}}$ of 55 ccMCTs with high intra- and interobserver reproducibility, taking approximately 10 minutes per tumor. $\bar{v_{v}}$ was associated with Patnaik and Kiupel grade, with cutoff values of 150 and 140 µm^3^ for G3 and HG, respectively. The slightly lower specificity and sensitivity of $\bar{v_{v}}$ values for Kiupel grade could be related to grading of HG ccMCTs, since these only need to fulfill one of the criteria proposed by Kiupel et al^14^; thus, karyomegaly and nuclear pleomorphism are less determinant. For instance, a tumor with 8 mitosis per 10/hpf would be classified as a HG ccMCT, independently of the nuclear size and/or variability.

Previous morphometric studies also found an association between Patnaik grade and both nuclear area and perimeter.^18,38,39^ In comparison with nuclear morphometry, $\bar{v_{v}}$ has the advantage of being “design-based,” meaning that nuclei are sampled with points and lines, independently of nuclear shape and orientation. Additionally, the PSI method provides a volume-weighted measurement of nuclear volume, meaning that nuclear size is favored. Therefore, larger nuclei have greater probability of being sampled.^11^ Particularly in poorly differentiated ccMCTs, the presence of lobulation and indentation is frequent, thus the elimination of assumptions about nuclear shape and orientation is required to perform statistically sound and reproducible estimations.

In terms of clinical outcome, $\bar{v_{v}}$ values were predictive of benign behavior, with 100% survival of dogs with ccMCT $\bar{v_{v}}\;$ <125 µm^3^. However, a $\bar{v_{v}}$ above 125 µm^3^ was associated with postsurgical progression of disease with 58% sensitivity and 100% specificity. Even though $\bar{v_{v}}$ was able to identify a good outcome, when a multivariate analysis was performed, the variable $\bar{v_{v}}$ was no longer statistically significant. Kiupel grading was the strongest predictor of clinical outcome, agreeing with previous evidence indicating its superior prognostic value.^13,27,40,41^ Interestingly, OC0 included 2 HG/G3 ccMCTs that corresponded to those with the highest $\bar{v_{v}}$ values. Similarly, OC1 included 1 G2/LG and 2 G2/HG ccMCTs with the lowest $\bar{v_{v}}$ values. The high association between histologic grade and $\bar{v_{v}}$ suggests that in order to identify outliers, additional prognostic factors which assess parameters other than nuclear pleomorphism are required. However, considering the small number of cases with poor outcome, a larger study is needed to refine this cutoff.

Nevertheless, some ccMCTs are diagnosed as HG based on the presence of karyomegaly, regardless of a low mitotic count. Kiupel et al define karyomegaly as “nuclear diameters of at least 10% of neoplastic mast cells vary by at least 2-fold”^13^ and this is likely poorly reproducible. In fact, scoring of nuclear pleomorphism has poor agreement between pathologists for other tumors.^7,28^ Considering that $\bar{v_{v}}$ was associated with high reproducibility, it could be added to Kiupel grading system for improving reproducibility in the assessment of nuclear pleomorphism.

## Supplemental Material

Supplemental Material, sj-pdf-1-vet-10.1177_0300985820985138 - Stereology in Grading and Prognosis of Canine Cutaneous Mast Cell Tumors Click here for additional data file. Supplemental Material, sj-pdf-1-vet-10.1177_0300985820985138 for Stereology in Grading and Prognosis of Canine Cutaneous Mast Cell Tumors by Mafalda Casanova, Sandra Branco, Inês Berenguer Veiga, André Barros and Pedro Faísca in Veterinary Pathology
