# Supplementary material for: Stereology in Grading and Prognosis of Canine Cutaneous Mast Cell Tumors
Source: Vet Pathol. 2021 Feb 12;58(3):483–90. doi: 10.1177/0300985820985138 (PMC8064533; doi:10.1177/0300985820985138)
Supplement: Supplemental Material, sj-pdf-1-vet-10.1177_0300985820985138 - Stereology in Grading and Prognosis of Canine Cutaneous Mast Cell Tumors [file sj-pdf-1-vet-10.1177_0300985820985138.pdf]

Supplemental Table S1. Animal raw data.

Case No	Mean nuclear volume (μm^3)[a]					Grade		Margins (cm)		Signalment			Progression of disease				
	#1	#2	#3	#4	Mean	Patnaik	Kiupel	Lateral	Deep	Age (years)	Sex	Breed	OC[b]	LR	LNM	DVM	Survival time
**1**	152.8	148.5	134.4	154.4	147.5	G2	LG	0.2	0.8	12	M	Mixed-breed	0	No	No	No	Alive
**2**	136.3	132.4	143.3	120.7	133.2	G2	LG	1.5	2	11	F	Beagle	0	No	No	No	Alive
**3**	104.1	102.0	113.4	77.5	99.2	G2	LG	1	2	11	F	Beagle	0	No	No	No	Alive
**4**	104.8	96.5	99.8	116.5	104.4	G2	LG	1.7	2	9	M	Mixed-breed	0	No	No	No	Alive
**5**	96.0	86.5	93.4	125.9	100.5	G2	LG	0.4	0.5	4	F	Labrador Retriever	0	No	No	No	Alive
**6**	86.4	81.0	87.4	106.9	90.4	G2	LG	0.3	0.3	6	M	Bassethound	0	No	No	No	Alive
**7**	112.4	134.5	155.0	130.8	133.2	G2	LG	0.5	0.5	12	M	Mixed-breed	0	No	No	No	Alive
**8**	108.6	116.9	124.9	101.6	113.0	G2	LG	1.5	2	4	F	Mixed-breed	0	No	No	No	Alive
**9**	80.0	108.9	111.2	76.8	94.2	G2	LG	0.2	0.3	7	F	Labrador Retriever	0	No	No	No	Alive
**10**	131.4	123.0	127.6	120.6	125.6	G2	LG	0.6	0.3	5	F	Golden Retriever	0	No	No	No	Alive
**11**	90.8	85.8	92.8	78.9	87.1	G2	LG	0.6	0.5	10	M	Bouvier Bernois	0	No	No	No	Alive
**12**	87.2	87.3	87.0	95.1	89.1	G2	LG	0.3	0.2	7	F	Mixed-breed	0	No	No	No	Alive
**13**	93.3	104.2	106.5	106.0	102.5	G2	LG	1	0.4	4	F	French Bulldog	0	No	No	No	Alive
**14**	109.4	110.1	106.1	76.8	100.6	G2	LG	3	1.3	11	M	Golden Retriever	0	No	No	No	Alive
**15**	156.9	107.4	137.5	136.0	134.5	G2	LG	2	0.5	9	M	Labrador Retriever	0	No	No	No	Alive
**16**	157.5	159.9	160.9	157.6	159.0	G2	LG	0.5	0.5	8	F	Labrador Retriever	0	No	No	No	Alive
**17**	124.2	134.3	127.6	137.5	130.9	G2	LG	0.4	0.4	10	F	Yorkshire Terrier	0	No	No	No	Alive
**18**	103.5	108.9	108.6	121.6	110.7	G2	LG	0.7	0.6	6	F	Weimanarer	0	No	No	No	Alive
**19**	123.3	115.5	144.5	113.1	124.1	G2	LG	0.2	0.1	2	M	French Bulldog	0	No	No	No	Alive
**20**	173.9	152.6	151.4	166.7	161.1	G2	LG	0.2	0.1	2	M	French Bulldog	0	No	No	No	Alive
**21**	91.8	95.2	91.9	119.7	99.6	G2	LG	3	2	10	F	Boxer	0	No	No	No	Alive
**22**	213.4	197.0	226.4	219.8	214.2	G3	HG	1.5	0.3	9	M	Mixed-breed	0	No	No	No	Alive
**23**	96.5	102.9	98.0	95.8	98.3	G2	LG	0.5	0.4	6	M	Pug	0	No	No	No	Alive
**24**	188.2	198.8	163.9	169.8	180.2	G3	HG	0	0.1	4	F	Bulldog	0	Yes	No	No	Alive
**25**	117.3	134.2	126.2	124.3	125.5	G2	HG	2	1	9	M	Boxer	1	Yes	Yes	No	1 month
**26**	165.1	186.3	168.9	164.9	171.3	G3	HG	1	0.6	9	F	Labrador Retriever	1	No	Yes	No	2 months
**27**	163.4	188.9	158.9	170.3	170.4	G3	HG	0	0	10	F	Labrador Retriever	1	Yes	Yes	No	1 month
**28**	181.9	198.3	175.1	188.7	186.0	G3	HG	0.2	0.2	10	M	Golden Retriever	1	Yes	No	No	3 months
**29**	141.8	158.8	136.3	140.6	144.4	G2	HG	1	0.5	10	F	Mixed-breed	1	Yes	No	Yes	17 months
**30**	105.1	139.2	128.5	137.6	127.6	G2	LG	2	1.6	12	F	Mixed-breed	1	No	No	Yes	8 months
**31**	110.1	92.2	92.2	103.4	99.5	G2	HG			No follow-up data or additional post-surgical treatment							
**32**	65.3	55.8	67.8	87.4	69.1	G2	LG			No follow-up data or additional post-surgical treatment							

**33**	222.2	229.8	238.6	230.9	230.4	G3	HG	No follow-up data or additional post-surgical treatment
**34**	165.3	170.2	196.7	170.6	175.7	G2	LG	No follow-up data or additional post-surgical treatment
**35**	141.4	128.7	139.4	133.1	135.6	G2	LG	No follow-up data or additional post-surgical treatment
**36**	258.2	272.4	259.6	233.4	255.9	G3	HG	No follow-up data or additional post-surgical treatment
**37**	276.3	286.3	261.4	299.2	280.8	G3	HG	No follow-up data or additional post-surgical treatment
**38**	167.0	170.0	186.6	176.6	175.0	G2	HG	No follow-up data or additional post-surgical treatment
**39**	142.6	151.6	127.3	123.8	136.3	G2	LG	No follow-up data or additional post-surgical treatment
**40**	109.1	110.9	97.7	89.5	101.8	G2	LG	No follow-up data or additional post-surgical treatment
**41**	149.8	150.1	177.9	157.2	158.7	G3	HG	No follow-up data or additional post-surgical treatment
**42**	239.1	188.5	216.6	205.5	212.4	G3	HG	No follow-up data or additional post-surgical treatment
**43**	116.1	92.9	114.3	96.0	104.8	G2	LG	No follow-up data or additional post-surgical treatment
**44**	115.9	119.5	130.2	131.8	124.3	G3	HG	No follow-up data or additional post-surgical treatment
**45**	144.1	156.7	152.4	159.3	153.1	G3	HG	No follow-up data or additional post-surgical treatment
**46**	113.7	122.0	109.9	135.4	120.2	G2	LG	No follow-up data or additional post-surgical treatment
**47**	92.1	109.3	106.2	100.8	102.1	G3	HG	No follow-up data or additional post-surgical treatment
**48**	35.2	34.5	39.0	45.8	38.6	G2	LG	No follow-up data or additional post-surgical treatment
**49**	46.3	55.5	56.1	81.6	59.9	G2	LG	No follow-up data or additional post-surgical treatment
**50**	157.7	182.0	162.3	168.8	167.7	G3	HG	No follow-up data or additional post-surgical treatment
**51**	96.3	111.9	118.4	104.3	107.7	G2	LG	No follow-up data or additional post-surgical treatment
**52**	365.2	380.1	338.5	369.5	363.3	G3	HG	No follow-up data or additional post-surgical treatment
**53**	119.2	119.6	96.2	105.4	110.1	G2	LG	No follow-up data or additional post-surgical treatment
**54**	165.1	180.5	198.9	167.2	177.9	G3	HG	No follow-up data or additional post-surgical treatment
**55**	114.0	117.3	108.6	126.6	116.6	G2	LG	No follow-up data or additional post-surgical treatment

Abbreviations: OC, outcome; LR, local recurrence; LNM, lymph node metastasis; DVM, distant visceral metastasis; G2, grade 2; G3, grade 3; LG, low-grade; HG, high-grade; M, male; F, female.

[a]Four individual measurements and mean.

[b]Survived(0), died or euthanized(1)
